# Plants’ bioactive secondary metabolites in the management of sepsis: Recent findings on their mechanism of action

**DOI:** 10.3389/fphar.2022.1046523

**Published:** 2022-12-16

**Authors:** Mohamad Fawzi Mahomoodally, Muhammad Zakariyyah Aumeeruddy, Lesetja Jan Legoabe, Stefano Dall’Acqua, Gokhan Zengin

**Affiliations:** ^1^ Institute of Research and Development, Duy Tan University, Da Nang, Vietnam; ^2^ Faculty of Natural Sciences, Duy Tan University, Da Nang, Vietnam; ^3^ Department of Health Sciences, Faculty of Medicine and Health Sciences, University of Mauritius, Réduit, Mauritius; ^4^ Centre of Excellence for Pharmaceutical Sciences (Pharmacen), North West University, Potchefstroom, South Africa; ^5^ Department of Pharmaceutical and Pharmacological Sciences, University of Padova, Padova, Italy; ^6^ Department of Biology, Faculty of Science, Selcuk University, Campus, Konya, Turkey

**Keywords:** sepsis, plants, secondary metabolites, phytochemicals, inflammation

## Abstract

Sepsis is a severe inflammatory response to systemic infection and is a threatening cause of death in intensive care units. In recent years, a number of studies have been conducted on the protective effect of natural products against sepsis-induced organ injury. However, a comprehensive review of these studies indicating the mechanisms of action of the bioactive compounds is still lacking. In this context, this review aimed to provide an updated analysis of the mechanism of action of plants’ secondary metabolites in the management of sepsis. Scopus, Science Direct, Google Scholar, and PubMed were searched from inception to July 2022. A variety of secondary metabolites were found to be effective in sepsis management including allicin, aloin, cepharanthine, chrysin, curcumin, cyanidin, gallic acid, gingerol, ginsenoside, glycyrrhizin, hesperidin, kaempferol, narciclasine, naringenin, naringin, piperine, quercetin, resveratrol, rosmarinic acid, shogaol, silymarin, sulforaphane, thymoquinone, umbelliferone, and zingerone. The protective effects exerted by these compounds can be ascribed to their antioxidant properties as well as induction of endogenous antioxidant mechanisms, and also *via* the downregulation of inflammatory response and reduction of biochemical and inflammatory markers of sepsis. These findings suggest that these secondary metabolites could be of potential therapeutic value in the management of sepsis, but human studies must be performed to provide strength to their potential clinical relevance in sepsis-related morbidity and mortality reduction.

## 1 Introduction

Sepsis is defined as a life-threatening organ dysfunction caused by a dysregulated host response to infection. It is a final common pathway to death from severe infectious diseases, such as bacterial bloodstream and lower respiratory tract infections, malaria, dengue, and systemic fungal infections. If not recognised early and managed on time, it can cause septic shock, multiple organ failure, and death (World Health Organization, 2020a). Patients who obtain severe COVID-19 complications have been also observed to be at a greater risk of death from sepsis (World Health Organization, 2020b). The incidence and mortality rate of sepsis differ considerably across areas, with the highest burden observed in sub-Saharan Africa, Oceania, South Asia, East Asia, and Southeast Asia (Rudd et al., 2020).

In 2017, 48.9 million cases and 11 million deaths were associated with sepsis globally, representing almost 20% of all deaths worldwide. Approximately 85.0% of sepsis cases and sepsis-associated deaths globally were reported in low- and middle-income countries. Almost half of all global sepsis cases were among children, with 20 million cases and 2.9 million global deaths in children estimated to be under 5 years of age (World Health Organization, 2020c). The updated review published in 2020 was based on 51 studies mostly from high-income countries (*n* = 46), and the estimate was a pooled incidence of 189 hospital-treated adult sepsis cases per 1,00,000 person-years and a mortality of 26.7%. Among hospital-born infants, hospital-acquired infections are responsible for an estimated 4%–56% of all deaths in the neonatal period which vary among geographical area (World Health Organization, 2020a). In the United States, the incidence of sepsis was doubled between 2000 and 2008 due to an increasing prevalence of non-communicable diseases in the aging population, together with the increase of antibiotic resistance and the high use of invasive procedures, immunosuppressive drugs, and chemotherapy. The expenses linked to sepsis management in the United States is greater than $20.3 billion every year (Dugar et al., 2020). Sepsis also frequently occurred from infections acquired in healthcare settings. In intensive care units patients, around one-half (48.7%) of sepsis cases were reported to be acquired in the hospital (World Health Organization, 2020a).

Antimicrobial resistance is a major burden in the management of sepsis. Sepsis patients with resistant pathogens were observed to show a higher risk of hospital mortality (World Health Organization, 2020c). A systematic review was carried out by Le Doare et al. (2015) on the antibiotic resistance rates in children with sepsis in resource-limited countries. In neonates, the median resistance of *Klebsiella pneumoniae* to ampicillin and cephalosporins in Asia was 94% and 84% respectively; while in Africa was 100% and 50%, respectively. Multidrug resistance to antibiotics such as ampicillin, chloramphenicol, and cotrimoxazole in Asia and in Africa was of 30% and 75%, respectively (Le Doare et al., 2015).

With regard to the pathophysiology of sepsis, the infection first causes an activation of the innate immune system resulting in the killing of bacteria by innate immune cells *via* numerous oxidative pathways (Armstrong et al., 2017). While bacterial infections continue to be the primary cause of pathogenic sepsis, viruses and fungi are also associated with a meaningful percentage of sepsis etiologies, especially among immunocompromised patients and those with other comorbidities. Antibacterial treatment has no effect on non-bacterial sepsis and is linked to increased mortality when mistakenly taken at high doses to treat fungal sepsis (Dolin et al., 2019). Normally the infectious insult is controlled with no progression to systemic process. Nonetheless, the extent of the infectious insult may sometimes cause a much more intense systemic activation. Consequently, activation of nitric oxide synthase leads to excess production of nitric oxide (NO), thereby raising oxidative stress, loss of vascular resistance, and resulting distributive shock. These oxidants damage cellular structures and biomacromolecules including DNA, mitochondrial cytochromes, signaling proteins, and membranes. The accumulating effects of dysregulation and cellular injury caused significant organ dysfunction (Armstrong et al., 2017).

The “Sepsis Six” is a series of interventions which can be undertaken during the treatment of severe sepsis condition; 1) administration of high flow oxygen, 2) take blood cultures, 3) provide broad spectrum antibiotics, 4) intravenous fluid challenges, 5) measurement of serum lactate and haemoglobin, and 6) measurement of hourly urine output (Uppu et al., 2015). In many low-income countries, important antimicrobials may not be accessible because of drug scarcity, high cost, or local import and regulation systems. Many hospitals do not have the required resources to establish current guidelines for sepsis management. Shortage of intravenous fluids, supplemental oxygen, simple positive pressure airway systems, and basic equipment to monitor health parameters such as pulse oximeters is also common (Rudd et al., 2018).

In recent years, a number of studies have been carried out considering the protective effect of some natural products, especially plants’ secondary metabolites, against sepsis-induced organ injury. Recently, a review was conducted on the mechanistic and therapeutic perspective in sepsis management with attention to some phytochemicals (Alikiaii et al., 2021). Plants provide a promising strategy in the treatment of sepsis *via* multiple actions including antimicrobial, anti-inflammatory or immunomodulatory properties, showing no or minimal unwanted secondary responses. Available drugs for sepsis management have not displayed promising effects so far, due to drug resistance, gastrointestinal distress, bleeding, hormonal imbalance, repetitive dosage regimen, and increasing medication cost (Usmani et al., 2020). So far, a comprehensive review of the studies investigating the mechanism of action of the plant bioactive compounds with a potential role in sepsis is still lacking. In this context, this review aimed to provide an updated analysis of the mechanism of action of secondary metabolites in the management of sepsis.

## 2 Methodology

The following databases were used for retrieving relevant articles: Scopus, Science Direct, Google Scholar, and PubMed. Articles were searched from inception to July 2022. We used the following keywords: “secondary metabolites + sepsis,” “phytochemicals + sepsis,” “phytocompounds + sepsis,” “plant compounds + sepsis,” “herbal compounds + sepsis,” “plant bioactive compounds + sepsis,” and “plant chemicals + sepsis.”

As for the screening procedure, full text investigation was performed when titles or abstracts provide information relevant to the study and the reference lists of the articles were further screened for related studies. Only articles in English and only studies on secondary metabolites with regard to sepsis were included. The following were excluded: 1) review articles, conferences, and case reports, 2) articles in non-English language, and 3) studies with abstract only accessible and without sufficient information.

**FIGURE 1:**
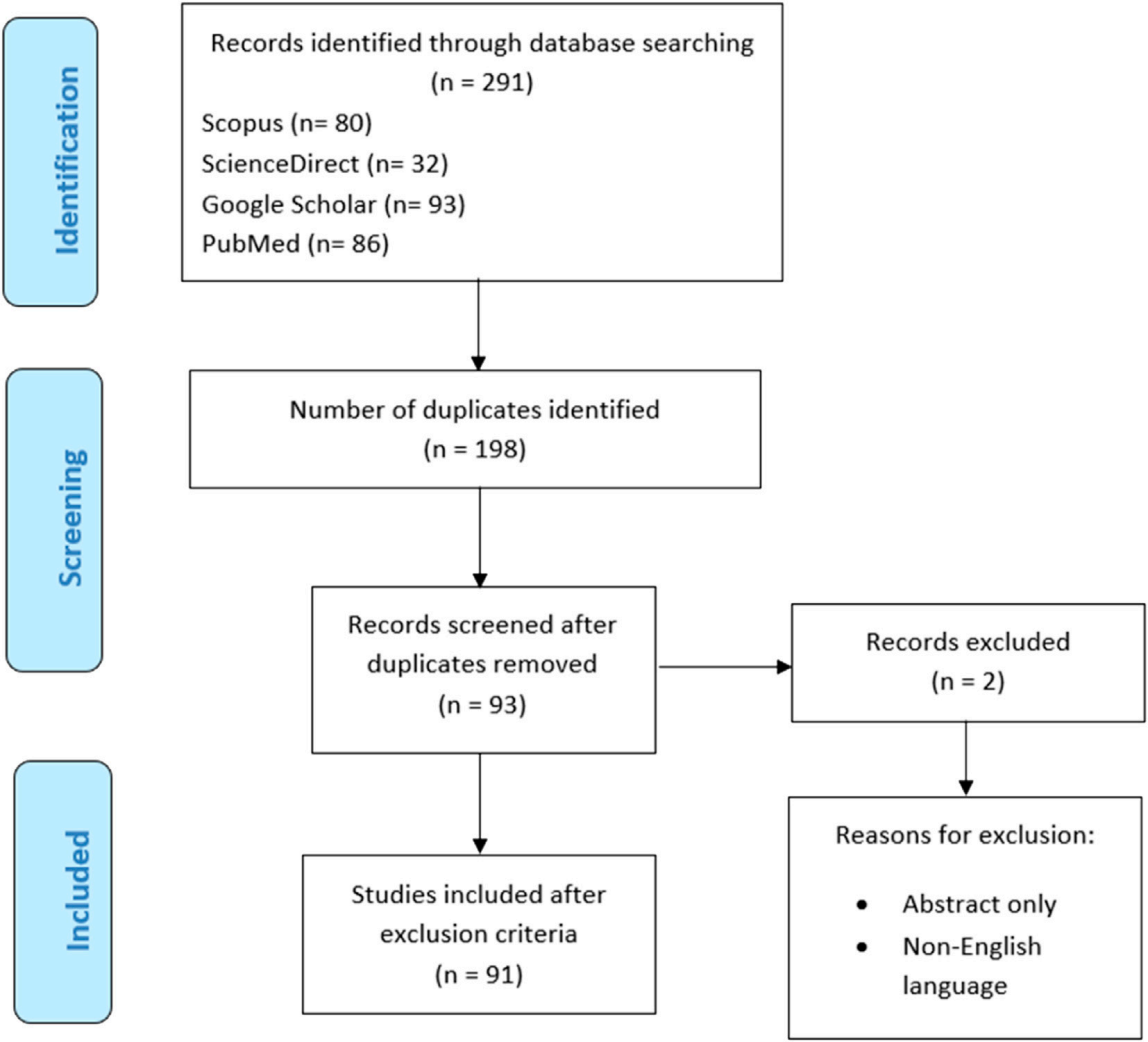
Flow chart used for systematic review.

A total of 291 studies were obtained from title screening in various databases (Scopus: *n* = 80; ScienceDirect: *n* = 32; Google Scholar: *n* = 93; PubMed: *n* = 86). The number of duplicated articles from these databases were 198 and, after removal of duplicates, 93 articles were left, of which two studies were excluded and 91 articles were included in the study (Figure 1).

## 3 Plants and their secondary metabolites

Plants make a huge and wide range of organic compounds, of which a major part do not seem to be involved directly in growth and development and are termed as secondary metabolites (Croteau et al., 2000). These compounds act as a natural defence system against attack of various microorganisms and environmental stresses. The functions of these compounds are in fact more than just providing protection because of their link to many biochemical pathways and possess a wide range of therapeutic applications (Kumar et al., 2022). On the other hand, the primary metabolites have an active role in the survival of species with regard to functions as photosynthesis and respiration. Secondary metabolites can be divided into three main groups: 1) terpenes (including plant volatiles, cardiac glycosides, carotenoids, and sterols), 2) phenolics (phenolic acids, flavonoids, coumarins, lignans, stilbenes, tannins, and lignin) and 3) nitrogen containing compounds (including alkaloids and glucosinolates) (Costa et al., 2012). These metabolites can be found in different parts of the plants such as leaf, stem, root, and bark. Secondary metabolites have a limited distribution in the plant kingdom, i.e., particular secondary metabolite are present in specific plant species or related group of species, while the primary metabolites are present throughout the plant kingdom (Anulika et al., 2016).

Drug discovery through combination of high throughput technology with selection of plants based on traditional information has been successful in finding novel chemical entities with potential as drug leads. Regardless of the development of combinatorial chemistry, the role of natural products in drug discovery remains very high. Among the 1,073 new chemical entities which had been approved between 1981 and 2010, only 36% were purely synthetic, while more than half were derived from nature (Atanasov et al., 2015). Some examples are; 1) anti-cancer agents, e.g., paclitaxel and its derivatives obtained in yew (*Taxus* L.) species, vincristine and vinblastine isolated from Madagascar periwinkle [*Catharanthus roseus* (L.) G. Don], and camptothecin and its analogs originally found in *Camptotheca acuminata* Decne; 2) galanthamine, a cholinesterase inhibitor which has been approved for the treatment of Alzheimer’s disease and was originally discovered in *Galanthus nivalis* L.; 3) khellin from *Ammi visnaga* (L.); Lam. which was the lead compound for development of chromoglicic acid, the sodium salt of which is utilised as mast cell stabilizer for managing allergy and asthma; 4) galegine from *Galega officinalis* L. which became the template for metformin synthesis and led to the subsequent development of biguanidine-type antidiabetic drugs; 5) papaverine from *Papaver somniferum* L. which aids in the development of the antihypertensive drug verapamil; 6) quinine isolated in the bark of Peruvian *Cinchona* L. species which was used in malaria treatment and also led to the synthesis of chloroquine and mefloquine, 7) the antimalarial drug artemisinin isolated in *Artemisia annua* L. (a TCM herb) in 1971 which led to the development of derivatives, including sodium artensunate or artemether, which are now commonly used for the treatment of malaria (Atanasov et al., 2015).

## 4 Secondary metabolites in the management of sepsis

**FIGURE 2:**
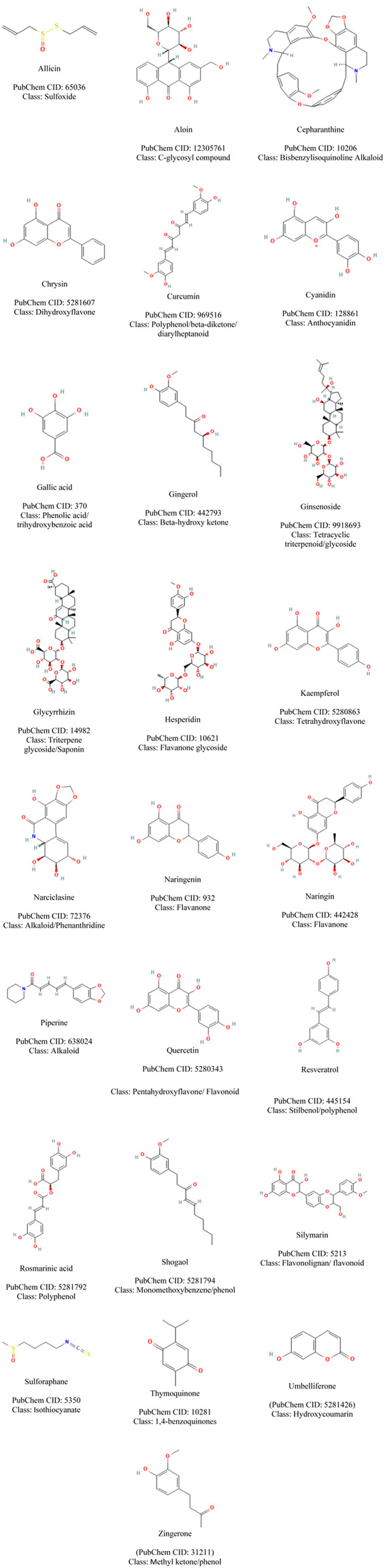
Plants’ secondary metabolites effective in the management of sepsis.

**FIGURE 3:**
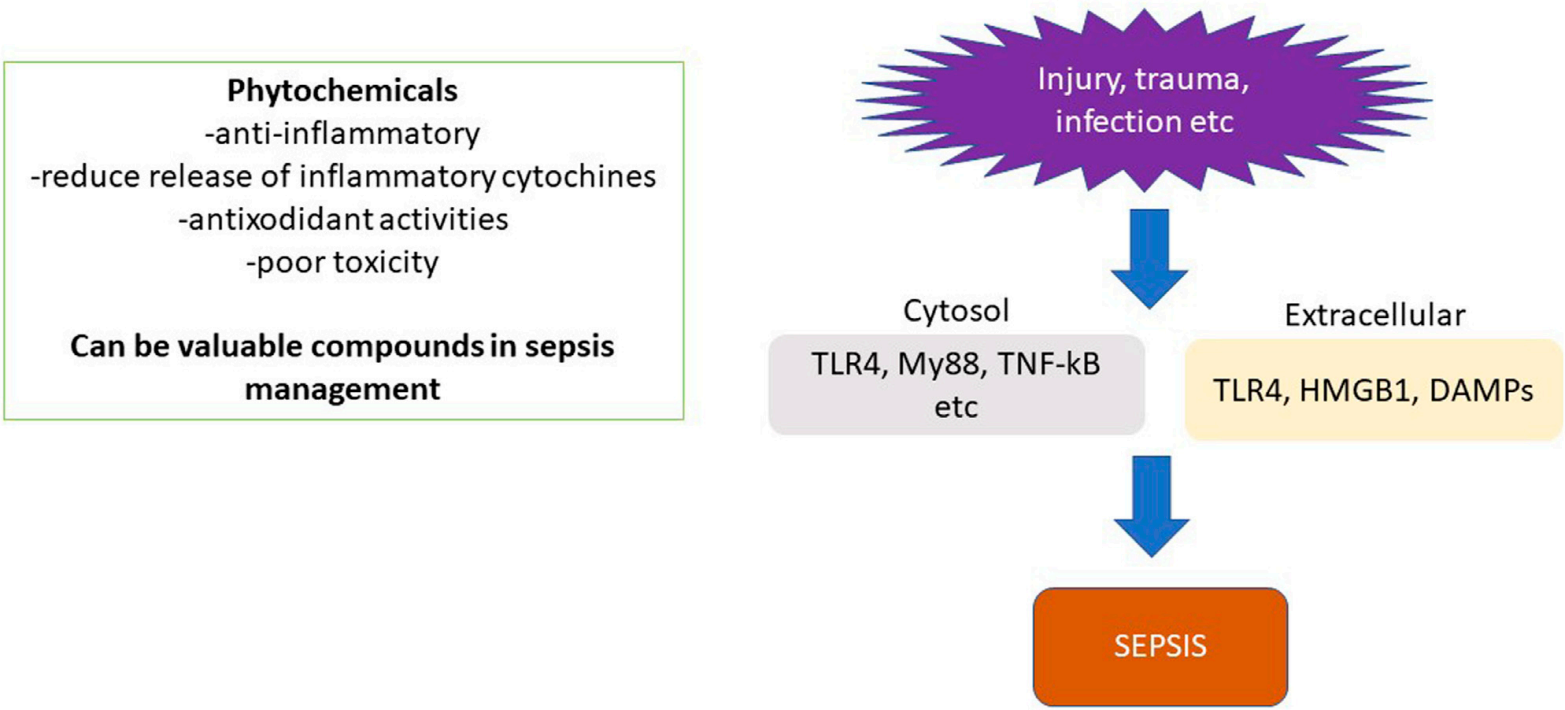
Molecular mechanism involved in sepsis.

**FIGURE 4:**
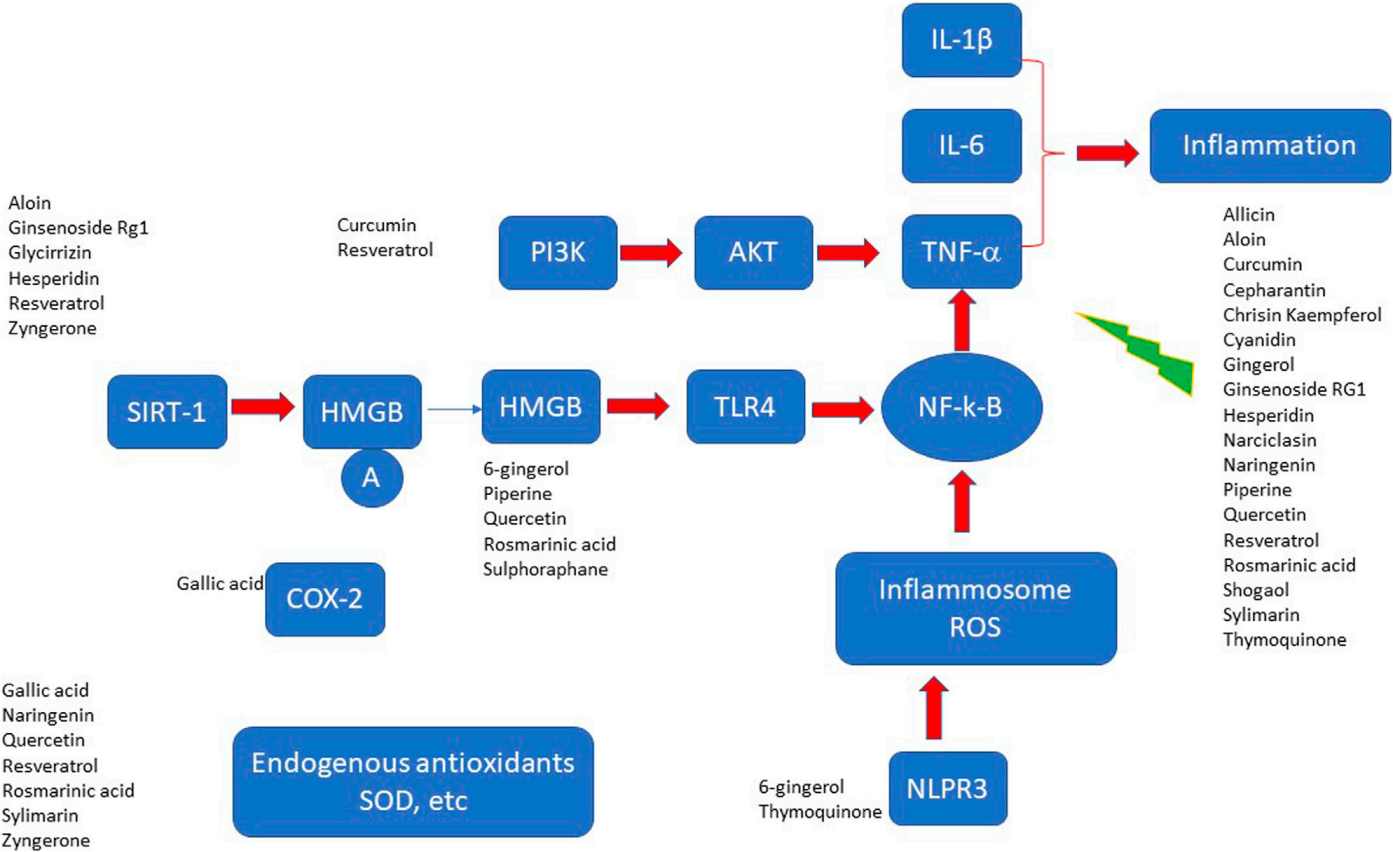
Potential targets of bioactive compounds from plants in sepsis.

A number of secondary metabolites have been investigated for their protective role in sepsis management and they belong to different chemical classes ranging from flavonoids, saponins, monoterpene, coumarins, and alkaloids showing the great varieties of chemical structures (Figure 2). The mechanism of actions is summarised in Table 1 and Figures 3, 4. As recently reviewed, the main target of actions are Toll-like receptor 4 (TLR4)/nuclear factor kappa-light chain-enhancer of activated B cells (NF-k-B) signal pathway. This pathway lead to hyper-expression of inflammatory cytokines (IL-1, IL-6, IL-8, and TNF-α). Besides high-mobility group box-1 (HMGB1), a damage associated molecular pattern (DAMPs), is also released to the circulation during the development of sepsis and phytochemicals targeted on this pathway are of high interest for their potential use in sepsis management (Alikiaii et al., 2021).

**TABLE 1:** Main mechanism of action of secondary metabolites in the management of sepsis.

Compound	Molecular mechanism of action	Reference
Allicin	•Downregulate TNF-α, IL-6, and IL-1β	Shen et al. (2019)
	•Suppressed TLR4, MyD88, NF-kB, and caspase protein activities	
Aloin	•Reduce HMGB1	Yang et al. (2019)
	•Activate SIRT1 and PI3K/Nrf2/HO-1 signaling pathway	
	•Inhibit LPS-induced release of TGFBIp	Lee and Bae (2021)
	•Inhibit NF-κB activation and reduced iNOS, IL-6 and TNF-α	Lee et al. (2019a)
	•Improve antioxidant defence system	
Cepharanthine	•Reduce IL-6, TNF-α, and nitrate/nitrite levels	Kudo et al. (2011)
	•Inhibiting leukocyte activation	Murakami et al. (2000)
	•Suppress NO production	Sakaguchi et al. (2007)
Chrysin	•Lower IL-1β, IL-10, TNF-α, IL-6, and MDA	Koc et al. (2020)
	•Upregulate Nfr2/Heme oxygenase 1 pathway	Xingyue et al. (2021)
Curcumin	•Reduce IL-1β, IL-6 and TNF-α	Zhong et al. (2016); Chen et al. (2018)
	•Suppress PI3K/AKT signaling pathway	
	•Inhibit Cyclic AMP-responsive element-binding protein/Caspase expression	
	•Reduce expression of TGF-β1 and SMAD3-dependent signaling pathway	Xu et al. (2013)
	•Up-regulate PPARγ	Siddiqui et al. (2006)
Cyanidin	•Lower TNF-α, IL-1β, IL-6, protein expression of cyclooxygenase-2 and production of prostaglandin E2	Ma et al. (2015); Yan et al. (2015)
	•Improve antioxidant defence system	
Gallic acid	•Suppress LPS-induced overproduction of prostaglandin E2, prostaglandin F2, leukotriene B4, thromboxane B2	Cheng et al. (2018)
	•Lower level of COX-2 and iNOS expression	
Gingerols	•Inhibit expression of NLRP3, IL-1β, and caspase-1	Hong et al. (2020)
	•Activate Nrf2	
	•Enhance bacterial clearance	Ju et al. (2021)
	•Reduce IL-1β	Zhang et al. (2020)
	•Block MAPK activation	
Ginsenosides	•Reduce IL-10, IL-6, and TNF-α, and enhanced bacterial clearance	Zou et al. (2013)
	•Reduce the expressions of TNF-α, IL-1β, and IL-6)	Li et al. (2017)
	•Suppress the expression of light chain 3-II and p62	
	•Upregulate SIRT1	Wang et al. (2019)
	•Activate AMPK pathway	Xing et al. (2017)
	•Decrease HMGB1 release	Lee et al. (2019)
	•Activate Akt/GSK-3β pathway through P2X purinoceptor 7 (P2X7) receptors	Liu et al. (2022)
	•Increase TUG1 expression and decrease expression of (miR)-200a-3p	Wu et al. (2021)
Glycyrrhizin	•Block HMGB1 signaling	Zhao et al. (2017b)
Hesperidin	•Attenuate expression of caspase-3, Bcl-2, TLR4, Hsp70, and MyD88 protein	Yuan et al. (2019)
	•Inhibit production of TNF-α, IL-6, and MCP-1	Liu et al. (2015)
Kaempferol	•Reduce IL-6, IL-1β, and TNF-α, nitrite and iNOS level, and downregulate mRNA expression of ICAM-1	Rabha et al. (2018)
	•Regulate MAPKs and NF-κB signaling pathways	Chen et al. (2012)
Narciclasine	•Lower systemic and local bacterial load and level of pro-inflammatory cytokines	Kingsley et al. (2020)
	•Inhibit c-Jun N-terminal kinase signaling pathway	Tang et al. (2021)
Naringenin	•Reduce Bax and raise Bcl-2 expression	Mu et al. (2019); Ye et al. (2020)
	•Inhibit AMPK and ACC phosphorylation, and reduce PGC1α expression	
Naringin	•Inhibit release of TNF-α and IL-6	Li et al. (2018)
	•Induce HO-1 expression in macrophages through AMPK, p38, and Nrf-2 signaling pathways	Gil et al. (2016)
	•Inhibit NF-κB nuclear translocation and activity of iNOS	Sun et al. (2019)
Piperine	•Decrease IL-1β and HMGB1 release	Liang et al. (2016)
	•Inhibit LPS-induced expression of type 1 interferon	Bae et al. (2010)
	•Decrease activation of signal transducer and activator of transcription (STAT)-1	
Quercetin	•Attenuate LPS-induced production of TNF-α and IL-1β	Chang et al. (2013)
	•Reduce HMGB1	Cui et al. (2019)
	•Induce macrophage M2 polarization	Zhu et al. (2019)
	•Inhibit LPS-induced iNOS expression and NO production	Angeloni and Hrelia (2012)
Resveratrol	•Suppress the overexpression of TNF-α, MMP-9, IL-1β, IL-6 and iNOS and increase IL-10	Li et al. (2013); Luo et al. (2020)
	•Inhibit NLRP3 expression	Sui et al. (2016)
	•Reduce leukocyte/platelet adhesion and E-selectin/ICAM-1 expression and increase SIRT-1 expression	Wang et al. (2015)
	•Induce activation of Nrf2 signaling	Wang et al. (2018)
	•Decrease oxidative DNA damage	Aydin et al. (2016)
	•Activate PI3K/AKT/mTOR pathway and inhibit NF-kB signaling pathway	Shang et al. (2019)
Rosmarinic acid	•Reduce DNA damage	Bacanlı et al. (2016)
	•Downregulate levels of TNF-α, IL-6, and HMGB1 protein	Jiang et al. (2009)
Shogaol	•Improve antioxidant defence system	Guo et al. (2021)
	•Suppress MAPK/NFκB induced inflammatory responses	
Silymarin	•Suppress production of IL-1β and prostaglandin E2	Kang et al. (2004)
	•Lower nitrite/nitrate, IL-6, and TNF-α levels	Al-Kadi et al. (2020)
	•Improve endogenous antioxidant mechanisms	
Sulforaphane	•Suppress release of HMGB1	Lee et al. (2017a)
	•Enhance Nrf2 and HO-1 protein expression	Zhao et al. (2017a)
	•Suppress LPS-induced secretion and mRNA expression of TGFBIp	
Thymoquinone	•Lower TNF-α, IL-1α, and IL-2 level	Alkharfy et al. (2011)
	•Inhibit the high levels of NLRP3, caspase-1, caspase-3, caspase-8, TNF-α, IL-1β, and IL-6	Guo et al. (2020)
	•Reduce phosphatidylinositide 3-kinase level	Liu et al. (2019)
Umbelliferone	•Antioxidant properties	Kutlu et al. (2020)
	•Reduce TNF-α mRNA expression	
Zingerone	•Inhibit NF-κB activation and reduce iNOS	Lee et al. (2017b)
	•Lower IL-6 and TNF-α	
	•Improve antioxidant defence system	
	•Decrease HMGB1 release	Lee et al. (2017b)
	•Suppress TGFBIp-mediated septic responses	Min et al. (2018)

### 4.1 Allicin

Shen et al. (2019) evaluated the *in vivo* effect and mechanisms of allicin, a volatile sulfide extracted from *Allium sativum* L. bulbs, in a sepsis-induced lung injury. Compared with lipopolysaccharide (LPS) group, lung injury was significantly improved by allicin which also down-regulated tumor necrosis factor α (TNF-α), interleukin (IL)-6, and IL-1β concentration. Allicin also significantly suppressed toll-like receptor (TLR4), MyD88, nuclear factor-kB (NF-kB), caspase-3 and caspase-9 protein activities in comparison to LPS group in lung tissues.

### 4.2 Aloin

Aloin, the main anthraquinone glycoside obtained from *Aloe* L. species, was observed to reduce high mobility group box 1 (HMGB1)-mediated septic responses and improved survival in septic mice by activating the sirtuin 1 (SIRT1) and PI3K/Nrf2/HO-1 signaling axis (Yang et al., 2019). Lee and Bae (2021) studied the inhibitory effects of aloin on Transforming growth factor *β*-Induced protein (TGFBIp)-mediated septic responses. Aloin inhibited LPS-induced release of TGFBIp and suppressed TGFBIp-mediated septic responses. The compound also suppressed TGFBIp-induced sepsis lethality and pulmonary injury.

Lee et al. (2019a) investigated the renal protective properties of aloin in a mouse model of sepsis. Aloin when administered intravenously at dose of 12.4 mg/kg significantly decreased the deleterious renal functions by cecal ligation and puncture (CLP), such as the elevated blood urea nitrogen, creatinine, and urine protein. Aloin also inhibited NF-κB activation and reduced inducible nitric oxide synthase (iNOS) and excess generation of nitric acid. The compound also lowered the plasma levels of IL-6 and TNF-α, decreased lethality due to CLP-induced sepsis, raised lipid peroxidation, and improved the antioxidant defence system through the restoration of superoxide dismutase (SOD), glutathione peroxidase (GPX), and catalase (CAT) levels in kidney tissues.

### 4.3 Cepharanthine

Cepharanthine, an alkaloid isolated from *Stephania cepharantha* Hayata, demonstrated effectiveness in managing systemic inflammatory response syndromes including sepsis by inhibiting the rise in LPS-induced cytokine levels (IL-6, TNF-α, and nitrate/nitrite levels) in rat serum. Histologic improvements were also observed with cepharanthine co-treatment while the LPS group displayed severe pathologic abnormalities including vacuolisation, dot necrosis, striped necrosis, and bridging necrosis, and inflammatory cells were observed adjacent to the necrotic tissue. *In vitro*, the compound also inhibited NF-kB activation by inhibition of the IKK pathway (Kudo et al., 2011). Cepharanthine also showed beneficial effect in the treatment of endotoxic shock in newborn rats by dose-dependently reducing the 24 h mortality of endotoxic shock. At 0.2 mg/kg dosage given by injection, it effectively lowered the mortality from 90% to 21% (Goto et al., 1991).

Sakaguchi et al. (2007) also found that cepharanthine confers protection to mice from endotoxin-induced lethal shock at 0.2–5 mg/kg, p.o. and against endotoxin/rhTNF-α-induced lethal shock at 1 mg/kg, i.p. *In vitro*, the compound (3 μg/ml) inhibited cell death in mouse L929 fibroblast. Also, cepharanthine suppressed NO production by endotoxin-stimulated Raw 264.7 mouse macrophage cells. Moreover, cepharanthine prevented LPS-induced pulmonary vascular injury by inhibiting leukocyte activation. Cepharanthine inhibited the functions of activated neutrophils including neutrophil elastase release, oxygen radical generation, and neutrophil aggregation, possibly by inhibiting the increase in intracellular free calcium concentration (Murakami et al., 2000).

### 4.4 Chrysin

Chrysin is a natural flavonoid, commonly present in a variety of plants but also in propolis, honey, and mushroom. Chrysin was found to lower the oxidative stress markers and cytokines in sepsis. Oral administration of chrysin, at doses of 50 and 100 mg/kg, significantly lowered the level of IL-1β, IL-10, TNF-α, IL-6, and malondialdehyde (MDA) in rats. Chrysin also increased the levels of SOD and CAT in tissues. Histopathological findings were also consistent with biochemical findings (Koc et al., 2020). Xingyue et al. (2021) also found that chrysin ameliorated sepsis-induced cardiac dysfunction through upregulating Nfr2/Heme oxygenase 1 pathway.

Chrysin and kaempferol can act synergistically improving the septic mice survival. The kaempferol/chrysin combination at 3 mg/kg enhanced the overall 7-day survival rate by 2-fold-up to 29%. Although the combination did not display significant antibacterial properties, the anti-inflammatory and antioxidant effects significantly improved the survival rate of septic mice (Harasstani et al., 2017).

### 4.5 Curcumin

Curcumin, the yellow pigment considered one of the primary bioactive substance in *Curcuma longa* L., was tested at doses of 20, 40, and 80 mg/kg, and found to alleviate LPS induced sepsis and liver failure in rats by suppression of oxidative stress-related inflammation *via* phosphatidylinositol 3-kinase/protein kinase B (PI3K/AKT) and NF-kB related signaling. Curcumin decreased the level of cytokines such as IL-1β, IL-6, and TNF-α, and also improved liver apoptosis by suppressing PI3K/AKT signaling pathway and inhibiting Cyclic AMP-responsive element-binding protein (CREB)/Caspase expression, while decreasing oxidative stress-associated protein expression (Zhong et al., 2016). Curcumin (50 and 200 mg/kg, i.p.) also protected against sepsis-induced acute lung injury in rat model by down-regulating the inflammatory cytokines TNF-α, IL-8, and macrophage migration inhibitory factor (MIF) levels. Interestingly, curcumin enhanced the survival rate of rats by 40%–50% (Xiao et al., 2012).

Treatment with curcumin significantly ameliorated inflammatory injury of the lung and kidney in septic mice. The compound (50, 100, and 200 mg/kg) enhanced the suppressive function of CD4^+^CD25^+^ regulatory T cells (Tregs) cells and raised the plasma levels of IL-10. It also inhibited the secretion of plasma TNF-α and IL-6 and improved the survival of septic mice (Chen et al., 2018). Xu et al. (2013) also investigated the effect of curcumin, intraperitoneal injection at 200 mg/kg, on sepsis-induced acute lung injury in a rat model. Real-time PCR and Western blot analysis revealed that curcumin significantly reduced the expression of TGF-β1 and SMAD3-dependent signaling pathway compared to control groups (*p* < .05).

Additionally, curcumin pre-treatment, 100 mg/kg intraperitoneal administration, modulated leukocyte and platelet adhesion and blood-brain barrier dysfunction in mice with CLP *via* P-selectin expression and also improved survival in mice with CLP (Vachharajani et al., 2010). Also, the anti-inflammatory effect of curcumin in an experimental model of sepsis was found to be mediated by upregulation of peroxisome proliferator-activated receptor-γ (PPARγ) (Siddiqui et al., 2006).

Furthermore, Yang et al. (2013) studied the protective effect of curcumin, administered by peritoneal injection (200 mg/kg/d, 3 days), against cardiac dysfunction in septic rat model. Curcumin significantly lowered the elevated levels of cardiac troponin I (cTn I) and MDA in plasma while raising the levels of SOD. Curcumin also enhanced the myocardial contractility by increasing the reduced Ejection Fraction (EF) and Fractional Shortening (FS) in septic rats induced by CLP. Myocardial inflammation and structure damage of myocardial cells were also alleviated by curcumin treatment.

### 4.6 Cyanidin

Cyanidin-3-O-glucoside is one of the most common anthocyanins naturally present in black rice, black bean, purple potato, and many fruits, such as red-skinned or red-fleshed apples, hawthorn, bilberries, cranberries, chokeberries, and lingonberries (Tan et al., 2019; Liang et al., 2021). This compound, injected intraperitoneally at doses 10 or 30 mg/kg, significantly improved survival rate and attenuated CLP-induced lung injury in rat model, including reduction of lung wet/dry weight ratio, protein leak, infiltration of leukocytes, and myeloperoxidase (MPO) activity. The phytochemical markedly reduced MDA content and raised SOD activity and glutathione level. The level of TNF-α, IL-1β, and IL-6 were also lowered, as well as protein expression of cyclooxygenase-2 and production of prostaglandin E2 in the lung. Cyanidin also increased protein expression of inhibitors of NF-kBa and decreased expressions of NF-kB p65 and p-p65 in the lung, thus inhibited the NF-kB-DNA binding activity (Yan et al., 2015).

Ma et al. (2015) also found that cyanidin-3-O-glucoside suppressed the production of pro-inflammatory cytokines (TNF-α, IL-6, and IL-1β) in cell supernatants and bronchoalveolar lavage fluid. Histopathologic examination revealed that cyanidin-3-O-glucoside pretreatment suppressed inflammatory cell infiltration, alveolar wall thickening, and interstitial edema in lung tissues. Western blot assay also revealed that cyanidin suppressed LPS-induced activation of NF-κB and mitogen-activated protein kinase (MAPK) signaling pathways in lung tissues.

### 4.7 Gallic acid

Gallic acid is a secondary metabolite present in most plants. Its distribution covers different families of the vegetable kingdom including Anacardiaceae, Fabaceae, and Myrtaceae (Fernandes and Salgado, 2016). Gallic Acid-L-Leucine conjugate was found to enhance survival and protect mice against LPS-induced inflammation and sepsis. The compound suppressed LPS-induced overproduction of prostaglandin E2, prostaglandin F2, leukotriene B4, thromboxane B2, and also significantly reduced the levels of cyclooxygenase-2 (COX-2) and iNOS expression and the plasma concentrations of proinflammatory lipid mediators in LPS-treated mice (Cheng et al., 2018).

Maurya et al. (2014) studied the prophylactic antioxidant potential of gallic acid in murine model of sepsis. Gallic acid (20 mg/kg, orally) significantly lowered MDA level in kidney, spleen, liver, and lungs of septic mice. A significant improvement was also observed in SOD activity of kidney and spleen when compared to septic mice.

### 4.8 Gingerols

Gingerols, major bioactive molecules from ginger, at 25 mg/kg injected intraperitoneally, was found to enhance survival of septic rats and attenuated sepsis-induced acute kidney injury by reducing renal disturbances, oxidative stress, and inflammatory response *via* a mechanism possibly related to increased production of dimethylamine and methylsulfonylmethane (Rodrigues et al., 2018). Moreover, Hong et al. (2020) investigated the effects of 6-gingerol (40 mg/kg, intragastric administration) on sepsis-induced liver injury. Mice pre-treated with 6-gingerol displayed less incidences of severe liver inflammation and mortality compared to untreated CLP groups. 6-gingerol treatment also inhibited the expression of pyroptosis-related proteins, such as NOD-like receptor protein 3 (NLRP3), IL-1β, and caspase-1. 6-gingerol was also observed to activate the nuclear factor-erythroid-2-related factor 2 (Nrf2) pathway *in vivo* and *in vitro*.

Additionally, 6-gingerol (20 mg/kg, orally) significantly improved sepsis development in CLP mice, as observed by a decrease in serum IL-1β. In bone marrow-derived macrophages and RAW264.7 cells, the compound attenuated pyroptosis and the release of caspase-1p20, HMGB1, IL-1β, IL-18 in response to ATP and LPS treatment. 6-Gingerol exhibited these effects by blocking MAPK activation (Zhang et al., 2020). 6-Gingerol-treated mice also displayed lower mortality in polymicrobial sepsis induced by CLP by enhancing bacterial clearance in the peritoneum, blood, and organs (liver, spleen, and kidney) while also suppressing the generation of TNF-α and IL-6 in TLR2 and/or TLR4-stimulated macrophages (Ju et al., 2021).

### 4.9 Ginsenosides

Ginsenoside Rg1, major active ingredient of *Panax ginseng* C.A. Mey., significantly improved survival in septic mice by lowering mortality rate (60%) in comparison to CLP group (30%). At doses of 20 mg/kg injected intravenously, Rg1 reduced IL-10, IL-6, and TNF-α, and enhanced bacterial clearance as evidenced by a reduction in bacterial counts in both blood and peritoneal lavage fluid when compared to control septic mice. Histologic examination revealed only minor abnormalities in lung and liver of Rg1-treated mice compared to CLP mice where lung injury occurred through alveolar wall thickening, neutrophils accumulation, and impaired alveoli. Also, Rg1 raised the neutrophil counts in peritoneal cavity while inhibiting lymphocyte apoptosis in thymus and spleen (Zou et al., 2013).

Rg1 improved the survival rate and provided protection against sepsis-associated learning and memory impairments (Morris water maze). The compound was administered at 40 and 200 mg/kg intraperitoneally, and found to attenuate brain histopathologic changes (hematoxylin and eosin staining), suppressed activation of Iba1, reduced the expressions of inflammatory cytokines (TNF-α, IL-1β, and IL-6), and also decreased neuronal apoptosis (cleaved caspase 3 activation) in hippocampus. Rg1 was found to suppress the expressions of light chain 3-II and p62 in hippocampus but not beclin 1 (Li et al., 2017).

Additionally, ginsenoside Rg1 relieved sepsis-induced lung injury *in vitro* and *in vivo*. The compound suppressed apoptosis rate of LPS-induced A549 cells, relieved mouse lung tissue damage, and increased survival rate. Rg1 also protected cells from LPS-induced intracellular reactive oxygen species (ROS) and suppressed the secretion of TNF-α and IL-6 and relieved cells from endoplasmic reticulum stress as evidenced by reduced expression of marker proteins *via* upregulating SIRT1 (Wang et al., 2019). Ginsenoside Rg3 was also observed to improve mitochondrial dysfunction by regulating autophagy in mitochondria *via* activation of AMP-activated protein kinase (AMPK) pathway, thus protecting cell and organ injuries caused by sepsis (Xing et al., 2017).

Moreover, ginsenoside Rh1 significantly decreased HMGB1 release in LPS-activated human umbilical vein endothelial cells (HUVECs). Rh1 inhibited the production of TNF-α, IL-6, activation of NF-κB and extracellular signal-regulated kinase (ERK) 1/2 by HMGB1. The compound inhibited HMGB1-mediated hyperpermeability and leukocyte migration in mice, and also decreased CLP-induced release of HMGB1, sepsis-related mortality and tissue injury *in vivo* (Lee et al., 2019). Other ginsenosides, Rk1 and Rg5, also showed suppressive effects on HMGB1-mediated septic responses (Kim et al., 2019).

Liu et al. (2022) also observed that Rg1, at 35 or 70 mg/kg injected intraperitoneally, activates the Akt/GSK-3β pathway through P2X purinoceptor 7 (P2X7) receptors to inhibit sepsis-induced cardiac dysfunction and mitochondrial dysfunction. In addition, Ginsenoside Rg3 increased taurine-upregulated gene 1 (TUG1) expression and decreased expression of microRNA (miR)-200a-3p to stimulate the silencing information regulator 1 (SIRT1)/AMPK pathway, thus enhancing autophagy to improve sepsis-induced liver injury and mitochondrial dysfunction (Wu et al., 2021).

### 4.10 Glycyrrhizin

Glycyrrhizin, known as glycyrrhizic acid, is a natural triterpene glycoside and a major active compound of *Glycyrrhiza glabra* L. (licorice) root. This compound was found to protect rats from sepsis by blocking HMGB1 signaling. The bioactive constituent attenuated the release and expression of HMGB1 and proinflammatory cytokines, while also blocking the interaction of HMGB1 with receptor for advanced glycation end products and toll-like receptor (TLR4) and suppressing the downstream MAPKs/NF-𝜅B signaling pathway. At a dose of 10 mg/kg, glycyrrhizin significantly decreased mortality caused by CLP (Zhao et al., 2017).

Likewise, Kim et al. (2015) found that glycyrrhizin decreased HMGB1 secretion in LPS-activated RAW 264.7 cells and endotoxemic mice by p38/Nrf2-dependent induction of heme oxygenase 1 (HO-1). Another study by Mollica et al. (2007) showed that glycyrrhizin inhibited the chemoattractant and mitogenic activities of HMGB1. In the same research, nuclear magnetic resonance and fluorescence studies revealed that glycyrrhizin binds directly to each of the two HMG boxes of HMGB1.

### 4.11 Hesperidin

Yuan et al. (2019) studied the protective effect of hesperidin, a flavanone glycoside abundant in citrus fruits (*Citrus* L.), at doses 10 and 20 mg/kg i.v., against sepsis CLP-induced lung injury in mice. Hesperidin was found to attenuate the partial pressure of arterial oxygen/fraction of inspired oxygen (PaO_2_/FiO_2_) ratio and lung injury score. The metabolite significantly reduced the level of proinflammatory mediators and attenuated the markers of oxidative stress. Administration of hesperidin also attenuated the expression of caspase-3, Bcl-2, TLR4, Hsp70, and MyD88 protein in the lung tissue.

Hesperidin was also observed to improve LPS-induced acute lung injury in mice when administered intragastrically at dose of 500 mg/kg. Production of proinflammatory cytokines and chemokine was inhibited, such as TNF-α, IL-6, and monocyte chemoattractant protein-1 (MCP-1). Also, the treatment inhibited the infiltration of macrophages and suppressed the expression and release of HMGB1 *in vivo* and *in vitro* (Liu et al., 2015).

### 4.12 Kaempferol

Kaempferol is a flavonoid found in many edible plants [e.g., tea (*Camellia sinensis* (L.) Kuntze), broccoli (*Brassica oleracea* var. *italica* Plenck), cabbage (*B. oleracea* var. *capitata* L.), kale (*B. oleracea* var. *sabellica* L.), beans (*Phaseolus vulgaris* L.), leek (*Allium porrum* L.), tomato (*Solanum lycopersicum* L.), strawberries (*Fragaria* × *ananassa* Duchesne ex Rozier) and grapes (*Vitis vinifera* L.)] (Calderón-Montaño et al., 2011). Kaempferol was synergically active with chrysin improving septic mice survival. The kaempferol/chrysin combination at dose of 3 mg/kg enhanced the overall 7-day survival rate by 2-fold-up to 29%. Although the combination did not display significant antibacterial effects, the anti-inflammatory and antioxidant effects significantly improved the survival rate of septic mice (Harasstani et al., 2017).

Rabha et al. (2018) evaluated the effect of kaempferol in sepsis-induced acute lung injury in mice with administration of 100 mg/kg orally. Kaempferol significantly reduced water content in lungs, as well as the cytokines IL-6, IL-1β, and TNF-α in plasma and lung tissue. Kaempferol also decreased the lung tissue nitrite level and iNOS level in septic mice, as well as downregulated the mRNA expression of intercellular adhesion molecule-1 (ICAM-1) and iNOS. Although no decrease in bacterial load was observed, kaempferol-treated mice displayed lesser infiltration of cells and more arranged alveolar structure.

Kaempferol (100 mg/kg, intragastrically) was also found to regulate MAPKs and NF-κB signaling pathways to attenuate LPS-induced acute lung injury in mice. Kaempferol inhibited LPS-induced alveolar wall thickness, alveolar hemorrhage and leukocytes infiltration in lung tissue, showing reduced MPO activity. Kaempferol increased SOD activity of lung tissue, which was reduced by LPS administration (Chen et al., 2012).

### 4.13 Narciclasine

Narciclasine is an isocarbostyril alkaloid discovered in *Narcissus* species (Amaryllidaceae) in 1967 (Fürst, 2016). The compound was found to improve outcome in sepsis among neonatal rats when administered at 3 mg/kg intraperitoneally. The compound lowered the plasma levels of S100A8/A9 and also suppressed its expression in the liver and lung. Narciclasine also reduced the systemic and local bacterial load as well as the generation of pro-inflammatory cytokines in plasma and organs (liver and lungs). Histopathological examinations revealed that narciclasine protected against organ damage associated with sepsis and enhanced the survival of neonatal rats. The compound also suppressed the phosphorylation of NF-κβ p65 and degradation of NF-κβ inhibitory protein alpha (Kingsley et al., 2020).

In addition, narciclasine was found to attenuate sepsis-induced myocardial injury *via* modulating autophagy. In this work the alkaloid was administered to mice at dose of 0.1 mg/kg by gavage. LPS-induced myocardial inflammation was attenuated and the compound also protected cardiac function and suppressed the expression of inflammatory cytokines in LPS-induced heart tissue. Also, narciclasine exhibited an inhibitory effect on the c-Jun N-terminal kinase (JNK) signaling pathway, and JNK activity was linked to narciclasine-induced autophagy and the subsequent protective effects during acute myocardial injury (Tang et al., 2021).

### 4.14 Naringenin

Naringin is a flavonoid found primarily in citrus species with especially high level found in grapefruit (*Citrus paradisi* Macfad.), bitter orange (*Citrus* × *aurantium* L.), and pomelo [*Citrus grandis* (L.) Osbeck] (Csuti et al., 2022). Administration of naringenin, a commonly occurring flavonoid aglycone, at 10 and 20 mg/kg to sepsis rats model significantly decreased the sepsis-induced apoptosis of kidney cells, and also reduced Bax and raised Bcl-2 expression. Naringenin also lowered the level of ROS and downregulated the expression of SOD, CAT, and ascorbate peroxidase (APX). The compound also reduced the level of urinary angiotensinogen in septic rats (Mu et al., 2019).

Ye et al. (2020) also investigated the role of naringenin in AMPK signaling pathway in LPS-induced septic cardiac dysfunction in mice. Naringenin treatment reduced IκB-α expression while raising the expressions of TNF-α, IL-6, and pNF-κB. The compound also significantly inhibited AMPK and ACC phosphorylation, reduced PGC1α expression and lowered the expression of complex I, complex II, and OPA1.

### 4.15 Naringin

Naringin, a well-known flavanone glycoside of grapes and citrus fruits formed by naringenin and the disaccharide neohesperidose, improved the survival rate of CLP mice and ameliorated sepsis-induced intestinal mucosal injury. The effects were observed with the compound at doses of 30 and 60 mg/kg. The compound improved impaired intestinal permeability, inhibited the release of TNF-α and IL-6, and raised the level of IL-10 in CLP mice and LPS-stimulated MODE-K cells. Using both *in vivo* and *in vitro* models, naringin showed increased expression of tight junction proteins ZO-1 and claudin-1 *via* RhoA/ROCK/NF-κB/MLCK/MLC signalling pathway (Li et al., 2018).

Gil et al. (2016) also studied the effect of naringin in CLP-induced sepsis mice. Naringin at dose of 200 mg/kg produced an anti-inflammatory effect by inducing HO-1 expression in macrophages through the AMPK, p38, and Nrf-2 signaling pathways. Naringin also improved sepsis-induced intestinal injury by modulating macrophage polarization *via* PPARγ/miR-21 axis (Li et al., 2021).

Naringin (50 and 100 mg/kg administered by oral gavage) also protected against sepsis-induced myocardial dysfunction by significantly reducing the levels of pro-inflammatory cytokines (TNF-α, IL-1β, and IL-6) and myocardial enzymes including creatine kinase (CK), lactate dehydrogenase (LDH), and aspartate aminotransferase (AST) induced by LPS. Naringin also inhibited NF-κB nuclear translocation and the activity of iNOS in H9c2 cardiomyocytes by activation of the PI3K/AKT signaling pathway (Sun et al., 2019).

### 4.16 Piperine

Piperine, an alkaloid present in black pepper (*Piper nigrum* L.), protected macrophages from pyroptosis and decreased IL-1β and HMGB1 release by suppression of ATP-induced AMPK activation, suggesting the potential of piperine to be a therapeutic agent against bacterial sepsis. Piperine dose-dependently inhibited ATP-induced pyroptosis, thus suppressing IL-1β or HMGB1 release in LPS-primed bone marrow-derived macrophages and J774A.1 cells. Moreover, *in vivo* findings revealed that piperine (20 mg/kg) decreased both peritoneal and serum IL-1β levels in mice intraperitoneally infected with *Escherichia coli*, suggesting the suppression of systemic inflammation and pyroptosis (Liang et al., 2016).

Additionally, piperine (1 or 5 mg/kg) was administered i.p. to mice and found to inhibit LPS-induced endotoxin shock, leukocyte accumulation and production of TNF-α, but not of IL-1β and IL-6. The compound inhibited LPS-induced expression of type 1 interferon (IFN) mRNA, as well as the levels of interferon regulatory factor (IRF)-1 and IRF-7 mRNA, and the phosphorylation and nuclear translocation of IRF-3. The bioactive compound also decreased activation of signal transducer and activator of transcription (STAT)-1, and inhibited the activation of STAT-1 in IFN-α/β-treated cells (Bae et al., 2010).

### 4.17 Quercetin

Quercetin, a natural flavonoid aglycone, is present in many products such as onion (*Allium cepa* L.), black tea [*Camellia sinensis* (L.) Kuntze], and broccoli (*B. oleracea* var. *italica* Plenck) (Kumar et al., 2017). In a mice model of lethal sepsis, quercetin significantly attenuated LPS-induced production of TNF-α and IL-1β in RAW264.7 macrophages, and also inhibited the LPS-stimulated phosphorylation of the inhibitors of κB kinase (IKKs), Akt, and JNK. Moreover, *in vivo* study showed that acute administration of quercetin (1, 10, 50, or 100 mg/kg injected intraperitoneally) decreased the lethality rate and circulating levels of TNF-α and IL-1β in C57BL/6J mice with endotoxemia induced by LPS (Chang et al., 2013).

Administration of quercetin intragastrically at a dose of 100 mg/kg was also found to be beneficial to acute lung injury by reducing the level of oxidative stress markers and raising the antioxidant enzyme activities in rat model of sepsis. The compound also lowered the serum levels of YKL-40 and periostin in the oxidative lung injury induced by the experimental sepsis model (Gerin et al., 2016).

Cui et al. (2019) showed that quercetin administration at the dosage of 15 and 20 mg/kg decreased tissue edema, congestion, and hemorrhage, while increasing the alveolar volume, and assist in maintaining the lung anatomy of septic rats. Significant reduction was observed in the ROS levels, as well as the activities and the expression of SOD, CAT, and APX. Quercetin also significantly reduced HMGB1 protein levels. Another study by (Zhu et al., 2019) revealed that intraperitoneal injection of 10 mg/kg quercetin protected murine sepsis by inducing macrophage M2 polarization *via* the TRPM2 dependent calcium influx and AMPK/ATF3 activation.

Additionally, Meng et al. (2016) studied the protective effect of quercetin on acute lung injury in rats with sepsis by administration of the compound at doses of 30 and 50 mg/kg. Quercetin showed improvement in arterial blood gases, lung water content, protein content, and cell counts in bronchoalveolar lavage fluid in comparison to control model group. The quercetin-treated groups also displayed lower serum ICAM-1 and macrophage inflammatory protein 2 (MIP2) expression compared to control group. In another study, intragastric administration of quercetin at a dose of 20 mg/kg also showed protective effect in hepatotoxicity induced by sepsis in rats (Doğukan et al., 2021).

Furthermore, Angeloni and Hrelia (2012) studied the protective effect of quercetin on LPS-induced septic rat cardiac dysfunction. Pretreatment of H9c2 cardiomyoblasts with 30 μM quercetin inhibited LPS-induced iNOS expression and NO production and prevented the oxidative stress resulted from unregulated NO production which generates peroxynitrite and other reactive nitrogen species. Moreover, quercetin pretreatment significantly counteracted apoptosis cell death and also inhibited the LPS-induced phosphorylation of stress-activated protein kinases (JNK/SAPK) and p38 MAP kinase.

### 4.18 Resveratrol

Resveratrol, a very well-known stilbenoid naturally present in dark grapes (*Vitis vinifera* L.) (Balanov et al., 2021), is highly considered as a nutraceutical with vasodilatory and antioxidant properties. The compound was found to suppress the overexpression of MMP-9, IL-1β, IL-6 and iNOS induced in LPS-induced sepsis mouse models and the TC-1 cell line in a dose-dependent manner. Resveratrol reduced pulmonary edema, improved lung function, and decreased pathological alterations in the septic mouse model when intraperitoneally injected at doses of 15 or 30 mg/kg (Li et al., 2013). Resveratrol also improved renal microcirculation, protected the tubular epithelium, and enhanced survival in a mouse model of sepsis-induced acute kidney injury (Holthoff et al., 2012).

In another study (Kolgazi et al., 2006) researchers used rats and induced sepsis. The animals with sepsis revealed a significant rise in MDA levels, MPO activity, and amount of collagen in lung and kidney tissues along with a decrease in glutathione levels. Microscopic examination showed severe destruction of regular morphology in both lung and kidney tissues. Septic rats also had higher serum TNF-α and LDH levels in comparison to the sham group. Resveratrol (30 mg/kg, i.p.) reversed these biochemical parameters and preserved tissue morphology as revealed by histological examination. Also, Sui et al. (2016) observed that resveratrol (30 mg/kg) showed protection against sepsis-associated encephalopathy and inhibited the NLRP3/IL-1𝛽 axis in microglia. The compound also attenuated the rate of apoptosis, decreased the number of iba-1 positive microglia in the hippocampus region, and also inhibited NLRP3 expression and IL-1𝛽 cleavage in a dose-dependent manner.

Wang et al. (2015) found that resveratrol administration (30 mg/kg injected intraperitoneally) reduced leukocyte/platelet adhesion and E-selectin/ICAM-1 expression with increased SIRT-1 expression in septic *ob/ob* and C57Bl/6 mice, and also reduced AC-p65 expression in HUVEC. The treated *ob/ob* mice also showed increased 7-day survival compared to the vehicle group. In another study by (Luo et al., 2020), CLP + resveratrol (30 mg/kg injected intraperitoneally) treated rats had greater survival rate (75.00%) compared to CLP group (41.67%). Resveratrol significantly reduced the level of serum creatinine and urea nitrogen, and relieved renal tubular swelling and luminal narrowing in CLP rats. Also, the compound significantly decreased the high expressions of GRP78, BiP, phosphorylated IRE1 and p65 proteins, as well as significantly lowered the levels TNF-α, IL-1β and IL-6 in CLP rats while increased that of IL-10.

Moreover, resveratrol alleviated sepsis-induced acute lung injury. Resveratrol treatment suppressed inflammation by reducing the production of TNF-α, IL-6, and IL-1β in LPS-stimulated MH-S cells, which was attributed to the inhibition of nuclear factor-κB, P38, and ERK signaling pathways. The compound also decreased LPS-induced apoptosis of MH-S cells by altering the unbalance of Bax/Bcl-2 and inhibition of LPS-induced autophagy (Yang et al., 2018).

Furthermore, resveratrol, intraperitoneally injected at 30 mg/kg, improved sepsis-induced acute kidney injury in rat model through the Nrf2 signaling pathway. Resveratrol decreased the LPS-induced inflammatory response in kidney cells *in vitro* and induced the activation of Nrf2 signaling (Wang et al., 2018). Resveratrol administration also significantly decreased the sepsis-induced oxidative DNA damage in the liver and kidney cells. In the resveratrol-treated sepsis rats (100 mg/kg, ip), the levels of MDA and TNF-α were lower while the level of reduced glutathione (GSH), SOD, and GPX activities were higher compared to septic group (Aydin et al., 2016).

In an *in vitro* study by (Silswal et al., 2018), resveratrol also downregulated biomarkers of sepsis by inhibiting proteasome’s proteases; chymotrypsin-like (predominantly LMP7), trypsin-like, and post-acidic protease activities. Also, resveratrol decreased LPS-induced inflammatory cytokine expression by reducing the translocation of NF-κB *via* an increase in inhibitor pIκBα.

In addition, resveratrol (intraperitoneal injection of 60 mg/kg) protected the myocardium in sepsis rat model *via* activation of the phosphatidylinositol 3-kinases (PI3K)/AKT/mammalian target of rapamycin (mTOR) pathway and inhibition of NF-kB signaling pathway (Shang et al., 2019). The study of An et al. (2016) also proved that intraperitoneal injection of 60 mg/kg resveratrol attenuated myocardial injury in sepsis rat model by reducing neutrophil accumulation, TNF-α expression, and myocardial apoptosis *via* activation of Sirt1 signaling.

### 4.19 Rosmarinic acid

Rosmarinic acid is an ester of caffeic acid with dihydroxyphenyl-lactic acid common in many herbs used in cuisine or as spice. This compound was first isolated from *Rosmarinus officinalis* L., since then, it has been successively found in more than 160 plants belonging to Lamiaceae, Boraginaceae, Apiaceae, etc. (Guan et al., 2022).

Rosmarinic acid significantly reduced DNA damage in the lymphocytes, livers, and kidneys of sepsis induced rats when administered at a dose of 100 mg/kg i.p. The compound reduced the level of MDA and increased GSH, SOD, and GPX activities in the livers and kidneys of the sepsis-induced rats. Plasma TNF-α level was also found to be lower in the rosmarinic acid-treated rats (Bacanlı et al., 2016).

Intravenous injection of rosmarinic acid alone or combined with imipenem treatment also decreased CLP-induced lethality in rats. The serum levels of TNF-α, IL-6, and HMGB1 protein were downregulated while the level of IL-10 was upregulated. Rosmarinic acid injection also ameliorated serum enzyme activities and MPO in lung, liver, and small intestine (Jiang et al., 2009).

### 4.20 Shogaol

Guo et al. (2021) evaluated the protective effect of 6-shogaol, a major bioactive component derived from ginger (*Zingiber officinale* Roscoe), on sepsis-induced liver injury. 6-shogaol, at dose of 20 mg/kg orally, was found to enhance the antioxidant defence system and suppressed MAPK/NFκB induced inflammatory responses. Transcriptomic analysis showed that the efficacy of 6-shogaol occurred *via* the regulation on Lcn2, Nos2, Ccl20, Cxcl1, Mmp9, Vcam1, Sele, Ptgs2, and Usp50 mRNA expression. Moreover, the protective effects of 6-Shogaol on renal injury caused by CLP was investigated in sepsis rat model. Oxidant molecules, MPO, and proinflammatory cytokine levels were reduced while antioxidant molecules were increased (Eraslan et al., 2020).

### 4.21 Silymarin

Silymarin is a mixture of flavolignans found in the fruits and seeds of milk thistle, *Silybum marianum* (L.) Gaertn. formed by silybin, silycristin, and silydianin. The compound showed protective effect against endotoxin-induced sepsis by displaying dose-dependent suppression of LPS-induced production of IL-1β and prostaglandin E2 (PGE2) in isolated mouse peritoneal macrophages and RAW 264.7 cells. Also, the mRNA expression of IL-1β and cyclooxygenase-2 was completely blocked while the LPS-induced DNA binding activity of NF-kB/Rel was also inhibited by silymarin in RAW 264.7 cells (Kang et al., 2004).

In the study of (Toklu et al., 2008), sepsis raised the level of serum TNF-α, IL-1β, IL-6, and LDH activity while reducing total antioxidant capacity. Also, CLP decreased the level of glutathione while increased that of MDA and MPO activity in both the lung and the brain tissues. Silymarin administration (50 mg/kg, p.o.), reversed these biochemical parameters and preserved tissue morphology as revealed by histological examination.

In addition, silymarin (100 mg/kg, i.p.) enhanced overall survival following sepsis compared to septic group (80% vs. 20%) in rat model, as well as improved hepatic and renal function parameters and decreased MDA, nitrite/nitrate, IL-6, and TNF-α levels. Silymarin also improved the endogenous antioxidant mechanisms by raising the level of GSH and reinforcement of SOD activity (Al-Kadi et al., 2020).

### 4.22 Sulforaphane

Sulforaphane is an organosulfur compound that is generated when the glucosinolate sulphoraphanin present in cruciferous vegetables such as broccoli (*B. oleracea* var. *italica* Plenck), Brussels sprout [*B. oleracea* var. *gemmifera* (DC.) Zenker], and cabbage (*B. oleracea* var. *capitata* L.), is hydrolysed by the enzyme myrosinase. Sulforaphane was found to suppress the release of HMGB1 and also downregulated HMGB1-dependent inflammatory responses in human endothelial cells. It also inhibited HMGB1-mediated hyperpermeability and leukocyte migration in mice, reduced sepsis-related mortality and pulmonary injury when administered at 0.26 or 0.39 mg/kg i.v. (Lee et al., 2017a). In the study of (Zhao et al., 2017a), supplementation of sulforaphane (5 mg/kg intraperitoneally) in rat sepsis model significantly enhanced NF-E2-related factor-2 (Nrf2) and HO-1 protein expression in the lungs. The molecule dose-dependently decreased pulmonary oxidative stress and attenuated lung injuries in sepsis. Moreover, sulforaphane suppressed LPS-induced secretion and mRNA expression of TGFBIp or CLP-induced TGFBIp secretion. The compound also inhibited TGFBIp-mediated hyperpermeability and activation of p38, and also protected against CLP-induced lethality and lung damage (Lee and Bae, 2017).

### 4.23 Thymoquinone

Alkharfy et al. (2011) studied the effect of thymoquinone, a monoterpene considered as the active compound of *Nigella sativa* L., against sepsis syndrome morbidity and mortality in mice. Thymoquinone decreased mortality by 80%–90% and ameliorated both kidney and hepatic biomarker profiles. The level of IL-1α with 0.75 mg/kg thymoquinone dose was 310.8 ± 70.93 compared to controls (1187.0 ± 278.64 pg/ml). Likewise, 0.75 mg/kg thymoquinone significantly decreased IL-10 compared to controls (2885.0 ± 553.98 vs. 5505.2 ± 333.96 pg/ml). Thymoquinone-treated group also displayed relatively lower levels of TNF-α and IL-2 (*p*-values = 0.1817 and 0.0851, respectively).

Guo et al. (2020) assessed the effect of thymoquinone on acute kidney injury induced by sepsis in BALB/c mice. The phytocompound, at dose of 50 mg/kg, lowered the level of serum creatinine and blood urea nitrogen and significantly inhibited the high levels of NLRP3, caspase-1, caspase-3, caspase-8, TNF-α, IL-1β, and IL-6 induced by CLP. Also, NF-κB protein level was significantly reduced in the thymoquinone-treated group compared to the CLP group.

In addition, thymoquinone significantly reduced intestinal histological alterations in sepsis-induced cardiac damage. Thymoquinone (50 mg/kg) inhibited plasma troponin-T levels and also inhibited p62, NLRP3, caspase-1, IL-1β, IL-18, IL-6, TNF-α, and MCP-1 expressions, while increasing beclin 1 and IL-10 level. The thymoquinone-treated group also displayed reduced phosphatidylinositide 3-kinase level compared to the CLP group (Liu et al., 2019).

Furthermore, the effect of thymoquinone was also studied on gram-negative bacteria-induced sepsis in mice. *Escherichia coli* inoculation caused an increase in the level of plasma cytokines such as TNF-α, IL-1, IL-2, IL-6, and IL-10. Likewise, the level of C-reactive protein, vascular endothelial growth factor, and mouse endothelial cell-specific molecule-1 (ESM-1) were increased in the septic mice. Administration of thymoquinone (1 mg/kg intraperitoneally) significantly downregulated the circulating concentrations of the inflammatory proteins. Most importantly, ∼75% of mice in the thymoquinone group survived at 96 h of observation in comparison to ∼8% in the untreated group (*p* = 0.0016) (Alkharfy et al., 2018).

Moreover, thymoquinone was found to prevent sequels of the multiple organ failure syndrome of sepsis *via* regulating the production of NO and its inflammatory sequela, and adjustment of vascular responsiveness (Alkharfy et al., 2015). Thymoquinone restored the erythrocyte deformability observed in CLP-induced sepsis in rats, showing more effectiveness when applied before the establishment of the sepsis (Bostanci et al., 2018). Ozer et al. (2017) found that thymoquinone intraperitoneally injected in rats at 1 mg/kg exhibited protective effects on mesenteric perfusion, contractile function of aorta as well as anti-inflammatory and antioxidative effects.

### 4.24 Umbelliferone

Kutlu et al. (2020) studied the effects of the coumarin umbelliferone isolated from *Ferulago pauciradiata* Boiss. & Heldr. on cecal ligation and puncture-induced sepsis model in rats. Different doses of umbelliferone (10, 20, and 40 mg/kg) were tested on oxidant-antioxidant level and mRNA expression level of inflammatory mediators such as TNF-α and IL-1 in septic rats. Comparison of the lung, kidney, and liver tissues of septic rats with those of the control group revealed that umbelliferone dose-dependently raised superoxide dismutase activity and glutathione levels while significantly lowered malondialdehyde levels. The 40 mg/kg dose displayed higher anti-oxidative properties than the 20 mg/kg and 10 mg/kg doses for all the evaluated parameters. Also, the TNF-α mRNA expression of the CLP + 40 mg/kg group was decreased to a level comparable to that of the control group. The findings suggest that umbelliferone intake may represent a promising treatment for the prevention of lung, kidney, and liver damage caused by septic conditions.

### 4.25 Zingerone

Lee et al. (2017b) investigated the renal protective properties of zingerone, a phenolic alkanone isolated from ginger, in a mouse model of sepsis. Zingerone treatment (0.72 mg/kg) inhibited NF-κB activation and reduced iNOS and excess production of nitric acid. Zingerone also lowered the plasma levels of IL-6 and TNF-α, reduced lethality due to CLP-induced sepsis, raised lipid peroxidation, and improved the antioxidant defence system by restoring the concentrations of SOD, GPX, and CAT in kidney tissues. Similarly in the study of (Wali et al., 2020), zingerone (150 mg/kg, orally) significantly restored plasma enzymes, antioxidant markers and attenuated proinflammatory cytokines and sepsis biomarker, thus prevented multi-organ and tissue damage in LPS-induced rats as confirmed by histopathological examination.

Another study by (Lee et al., 2017b) showed that zingerone significantly decreased HMGB1 release in LPS-activated HUVECs *via* SIRT1-mediated deacetylation of HMGB1. The phytochemical suppressed the production of TNF-α and IL-6 and the activation of NF-κB and ERK 1/2 by HMGB1. Also, HMGB1-mediated hyperpermeability and leukocyte migration were inhibited in mice by zingerone (0.36 or 0.72 mg/kg, i.v.).

Zingerone treatment (25 and 50 mg/kg) in septic mice also attenuated neutrophil extracellular traps (NETs) formation and inflammatory response *via* Nrf2-mediated ROS inhibition, thereby providing a therapeutic strategy against sepsis-induced injury (Zhu et al., 2022). Furthermore, the suppressive effects of zingerone on TGFBIp-mediated septic responses was evidenced by (Min et al., 2018). The compound inhibited both LPS and CLP-mediated release of TGFBIp, expression of TGFBIp receptor (integrin αvβ5), and TGFBIp-mediated barrier disruption. Zingerone also reduced human neutrophil adhesion and migration toward HUVECs, and reduction in TGFBIp-induced mortality in mouse model, tested at doses 0.36 or 0.72 mg/kg intravenously.

## 5 Conclusion

Different plants’ secondary metabolites have been evaluated in several *in vivo* and *in vitro* models and the results show that these compounds can induce protection in sepsis experimental animals and reduce sepsis-induced mortality. These compounds exhibit protective effect on cardiac, lung, liver, and kidney against sepsis-induced acute organ damage, and several studies showed the ability of these phytochemicals to modulate cytokines related to inflammation such as IL-6, IL-1β, and TNF-α. Some of these phytochemicals such as naringin and naringenin (flavanones), quercetin and kaempferol (flavonols), crysin, curcumin, resveratrol and the cyanidin-3-o-glucosides (flavones) are very diffused in the vegetable food suggesting their safe use. Also, the other compounds that have been evaluated have been isolated from spices or culinary herbs supporting their use in food.

Results indicate multiple mechanism of action of these compounds but for many of these phytoconstituents, the protective effects can be attributed to bacterial clearance, antioxidant properties as well as induction of endogenous antioxidant mechanisms, and also *via* the downregulation of inflammatory response and reduction biochemical and inflammatory markers of sepsis. These findings suggest that these secondary metabolites could be of potential therapeutic value in the management of sepsis, but require that human studies be performed to provide further evidence to their potential clinical efficacy and safety in reducing sepsis-related morbidity and mortality.

## Abbreviations

AMPK, AMP-activated protein kinase; CAT, catalase; CLP, cecal ligation and puncture; GPX, glutathione peroxidase; HMGB1, high mobility group box 1; HO-1, heme oxygenase 1; HUVEC, human umbilical vein endothelial cells; ICAM, intercellular adhesion molecule; IL, interleukin; iNOS, inducible nitric oxide synthase; IRF, interferon regulatory factor; JNK, c-Jun N-terminal kinase; LDH, lactate dehydrogenase; LPS, lipopolysaccharide; MAPK, mitogen-activated protein kinase; MDA, malondialdehyde; MPO, myeloperoxidase; NF-kB, nuclear factor-kB; NLRP3, NOD-like receptor protein 3; NO, nitric oxide; Nrf2, nuclear factor-erythroid-2-related factor 2; ROS, reactive oxygen species; SIRT1, sirtuin 1; SOD, superoxide dismutase; TGFBIp, transforming growth factor *β*-induced protein; TLR, toll-like receptor; TNF-α, tumor necrosis factor *α*.
